# The risk of cardiovascular disease in women after miscarriage, stillbirth, and therapeutic abortion: a protocol for a systematic review and meta-analysis

**DOI:** 10.1186/s13643-020-01444-0

**Published:** 2020-10-07

**Authors:** Charlotte Muehlschlegel, Harry Kyriacou, Abdulrahman Al-Mohammad, Lowri A. Foster-Davies, Fiona Simmons-Jones, Clare Oliver-Williams

**Affiliations:** 1 School of Clinical Medicine, University of Cambridge, Cambridge, England; 2 Essex County Council, County Hall, Chelmsford, Essex CM1 1QH England; 3 Homerton College, University of Cambridge, Cambridge, England; 4 Cardiovascular Epidemiology Unit, Department of Public Health and Primary Care, University of Cambridge, Cambridge, England

**Keywords:** Miscarriage, Stillbirth, Abortion, Cardiovascular disease, Coronary heart disease, Stroke, Systematic review, Meta-analysis, Protocol

## Abstract

**Background:**

Cardiovascular disease (CVD) is the leading cause of death in women, responsible for approximately a third of all female deaths. Pregnancy complications are known to be associated with a greater risk of incident CVD in mothers. However, the relationships between pregnancy loss due to miscarriage, stillbirth, or therapeutic abortion, and future maternal cardiovascular health are under-researched. This study seeks to provide an up-to-date systematic review and meta-analysis of the relationship between these three forms of pregnancy loss and the subsequent development of CVD.

**Methods:**

This systematic review will follow the Preferred Reporting Items for Systematic Review and Meta-Analysis checklist (PRISMA) and the Meta-analyses Of Observational Studies in Epidemiology (MOOSE) Checklist. A systematic search will be undertaken using publications identified in MEDLINE (PubMed), Scopus, Web of Knowledge, the CINAHL Nursing Database, and the Cochrane Library. The eligibility of each publication will be determined by predefined selection criteria. The quality of the included studies will be rated using the Newcastle-Ottawa Scale. Pooled measures of association will be computed using random-effects model meta-analyses. Between-study heterogeneity will be assessed using the *I*^2^ statistic and the Cochrane *χ*^2^ statistic. Small study effects will be evaluated for meta-analyses with sufficient studies through the use of funnel plots and Egger’s test.

**Discussion:**

The results of this systematic review will discuss the long-term risks of multiple types of cardiovascular disease in women who have experienced miscarriage, stillbirth, and/or therapeutic abortion. It will contribute to the growing field of cardio-obstetrics as the first to consider the full breadth of literature regarding the association between all forms of pregnancy loss and future maternal cardiovascular disease.

**Systematic review registration:**

PROSPERO registration number [CRD42020167587]

## Background

Cardiovascular disease (CVD) encompasses a number of different conditions affecting the cardiovascular system, such as stroke and myocardial infarction (MI) [1, 2]. CVD presents one of the largest global public health challenges, causing more deaths globally than any other cause; approximately 32% of global deaths and a third of all female deaths in 2017 [1, 3–5]. It is also the leading cause of non-communicable disability-adjusted life years lost [6]. The majority of the global health burden, of both death and disability, associated with CVDs can be attributed to two main subtypes: coronary heart disease (CHD), also known as ischemic heart disease, and stroke [7–9].

Given that most CVD-related deaths and disabilities are due to modifiable or preventable risk factors, it remains a top priority for public health and preventative measures globally. However, CVD research has historically been focused on men—with recent textbooks still describing the common male CVD presentation as the standard presentation [10]. This is beginning to change. The recent identification of female-specific factors associated with a greater CVD risk provides the potential to implement effective and targeted preventative measures to decrease disease burden at an individual and population level [11].

Female-specific risk factors known to be associated with greater CVD risk include early menopause, early menarche, and hysterectomy [12]. Changes that occur during pregnancy, such as pre-eclampsia, gestational diabetes, and preterm birth, have also been linked to greater CVD risk in women [11].

Pregnancy loss through miscarriage is, sadly, a common pregnancy complication. Up to 60% of all pregnancies end in miscarriage [13, 14], while 2% of British women annually have a therapeutic abortion (henceforth referred to as abortion) [15] and 1.8% of babies are stillborn worldwide (0.3% in developed countries) [16]. Prior studies have indicated a greater CVD risk for women who have repeated miscarriages or a stillbirth [11, 12, 17]. However, previous systematic reviews on this topic do not incorporate the most up-to-date publications, have disparate inclusion and exclusion criteria, and between-study heterogeneity [11, 12]. As a result, there is a lack of clarity about (1) the absolute and relative magnitude of CVD risk after miscarriage and stillbirth, (2) the risk associated with recurrent pregnancy loss, and (3) the risk of different subtypes of CVD, as prior reviews at most evaluated CVD, CHD, and stroke. Furthermore, no reviews evaluated CVD risk after abortions, although this provides a natural control for the miscarriage analyses, as both result in cessation of a pregnancy, primarily in the first 12 weeks of gestation.

To address these knowledge gaps, a review summarizing this area of research is needed, and a systematic review protocol was developed to assist the identification, evaluation, and interpretation of all relevant research. All authors followed this protocol during the literature review and meta-analysis of published studies. Existing guidelines for meta-analyses, systematic reviews, and protocols were consulted (MOOSE, Cochrane Handbook, Guidance on the Conduct of Narrative Synthesis in Systematic Reviews, PRISMA, and PRISMA-P) in the development of this protocol [18–22].

This study seeks to provide an up-to-date summary of the evidence for the long-term CVD risk for women who have experienced pregnancy loss compared to women who have not. It serves as an update to, and expansion of, a similar systematic review and meta-analysis carried out in 2011 [17], which focused on the association between miscarriage and future maternal CVD specifically. This review has wider inclusion criteria, through the addition of stillbirth and therapeutic abortion as exposures and added sensitivity analyses to increase methodological robustness. All methodological changes have been designed a priori and are clearly outlined in this protocol. PRISMA and MOOSE checklists will be completed for this review [18, 21].

## Methods/design

This review protocol followed the Cochrane Handbook; PRISMA-P, PRISMA, and MOOSE guidelines; and Guidance on the Conduct of Narrative Synthesis in Systematic Reviews [18–22], which guide the preparation and reporting of protocols, systematic reviews, and meta-analyses. They collectively led to the development of systematically defined inclusion and exclusion criteria, an analytical framework and a structure for writing up the protocol. Any changes from these protocols, along with the justification for these changes, are listed below. Table 4 in Appendix displays the completed PRISMA-P checklist.

### Inclusion criteria

Publications will be eligible for inclusion in this review if they meet the following requirements:

Participants: Women > 15 years of age with no major comorbidities and no CVD before pregnancy.

Exposures and comparators: Any of the following:

1. Miscarriage: pregnancy loss that is spontaneous, involuntary, and occurs before 28 weeks of gestation prior to the 1st of October 1992, and before 24 weeks after this date [23]. Studies which do not report a definition will still be included.
  Comparator: women who have not experienced or reported any miscarriages.
2. Stillbirth: pregnancy loss that occurs at 28 or more weeks of gestation prior to the 1st of October 1992, and after 24 weeks after this date [24].
  Comparator: women who have not experienced or reported any stillbirths.
3. Abortion: the intentional termination of a pregnancy, whether viable or non-viable, so that it does not result in the birth of a child [25]. No distinction will be made between medical abortion and surgical abortion in this systematic review, nor will distinctions be made on the grounds for abortion (e.g., problems identified in the embryo vs abortion related to the pregnant women’s health)
  Comparator: women who have not experienced or reported any abortions.

Outcomes: any of the following: (1) total CVD, (2) CHD, (3) total stroke, (4) ischemic stroke, (5) hemorrhagic stroke, (6) myocardial infarction, and (7) transient ischemic attack.

Study design: Cohort of case-control studies, which include a measure which includes a measure of association or the raw data to calculate one.

### Exclusion criteria

Publications which solely used neonatal deaths or ectopic pregnancies as exposures will be excluded in order to minimize heterogeneity in exposure measures. Editorials, protocols, and conference proceedings will not be included in the review due to the lack of methodological details. Publications evaluating only CVD risk factors, and those combining different exposure groups together (e.g., miscarriage and stillbirth as a single exposure group) will not be eligible. Papers will not be excluded based on the grounds for abortion (e.g., health of the embryo vs health of the pregnant woman), nor the medical treatment received for pregnancy loss (e.g., medical vs surgical), nor the gestational duration of the pregnancy when it ended.

To ensure a high level of interrater consistency, a consistency check will be carried out prior to the screening of results using the kappa statistic. This check will be conducted through the application of the selection criteria by 4 reviewers independently to a 20% random sample of the results of the systematic searches at the title/abstract stage. Following this, the criteria will be further refined, if required, in order to support consistent interpretation between members of the review team.

### Information sources

The following databases will be systematically searched for relevant publications: MEDLINE (through PubMed), Scopus, Web of Knowledge, the CINAHL Nursing Database, and the Cochrane Library. To optimize this systematic literature search, the reference lists of identified studies and related reviews will also be manually screened for other pertinent publications meeting our inclusion criteria.

### Search strategy

To ensure the search strategy is as comprehensive as possible, a search of MEDLINE will be performed using an initial set of search terms created from MeSH & thesaurus terms (Table 1). The titles and abstracts from this search will be assessed for any additional relevant search terms. If any are identified, they will be included in the final, comprehensive search strategy.

**Table 1:** Search terms and number of articles found from a preliminary PubMed search

Step	Search term	Articles found (*n*)^a^
#1	“Spontaneous Abortion” OR “Habitual Abortion” OR “Recurrent Abortion” OR “Recurrent Miscarriage” OR “Habitual Miscarriage” OR “Miscarriage” OR “Foetal Death” OR “Fetal Death” OR “Pregnancy Loss” OR “therapeutic abortion” OR “stillbirth^a^” OR “still birth” OR “still-birth” OR “still born^a^” OR “abortion^a^” OR “induced abortion^a^”	49,041
#2	“Cardiovascular Disease” OR “Coronary Artery Disease” OR “Myocardial Infarction” OR “Heart Attack” OR “Coronary Heart Disease” OR “Ischemic Heart Disease” OR “Ischaemic Heart Disease” OR “Stroke” OR “Transient Ischemic Attack” OR “Transient Ischaemic Attack” OR “Vascular Accident” OR “Apoplexy” OR “cerebrovascular disease^a^” OR “TIA” OR “CVA” OR “cva” OR “CVD” OR “cardio-vascular disease” OR “Coronary Artery Bypass Graft”	769,180
#3	#1 AND #2 (filtered to humans)	421

^a^Preliminary search conducted on May 7, 2019

No restrictions on the publication period, geography, or language of the articles will be included as part of the search methodology. This is in order to ensure that the results are as complete as possible. Searches will not be limited to titles or abstracts; medical search headings and open-text fields will be used in the database searches to return publications. The search in MEDLINE will be limited to humans.

Final search terms for the exposure (e.g., pregnancy loss) and outcome (e.g., CVD) are shown in Table 2. While this review in part updates a previously published systematic review and meta-analysis [17] which included results published prior to December 2011, additional search terms have also been included. Therefore, there will be no restrictions on the publication period of the search to ensure all relevant results are identified.

**Table 2:** Search terms used in the full systematic literature search

**Exposure**	“Spontaneous Abortion” “Habitual Abortion” “Recurrent Abortion” “Recurrent Miscarriage” “Habitual Miscarriage” “Miscarriage” “Foetal Death” “Fetal Death” “Pregnancy Loss” “therapeutic abortion” “still birth*” “still-birth*” “stillbirth*” “still born*” “abortion*” “induced abortion*”
**Outcome**	“Cardiovascular Disease” “Coronary Artery Disease” “Myocardial Infarction” “Heart Attack” “Coronary Heart Disease” “Ischemic Heart Disease” “Ischaemic Heart Disease” “Stroke” “Transient Ischemic Attack” “Transient Ischaemic Attack” “Vascular Accident” “Apoplexy” “cerebrovascular disease*” “TIA” “CVA” “cva” “CVD” “cardio-vascular disease” “Coronary Artery Bypass Graft”

Non-underlined text: search terms from 2013 review and meta-analysis

Underlined text: additional search terms included in this review

### Study selection

A database compiled using CADIMA, a tool supporting the conduct and reporting of systematic reviews [26] will be used for screening eligible publications. Following deduplication, studies will be screened for inclusion using a hierarchical review methodology: all titles and abstracts will be reviewed, and the full texts and supporting data of potentially relevant publications identified at this stage will then assessed.

Each study will be independently screened for eligibility by two members of the review team, against predefined inclusion criteria. Any disagreements will be resolved through discussion between the two reviewers; however, if consensus cannot be reached, a third reviewer will be consulted. All publications selected for inclusion in this study will be approved by a senior investigator. The numbers of publications reviewed, full-text studies retrieved, and studies excluded will be reported using the PRISMA flow chart.

If multiple publications are found to use the same primary data from the same individuals, the publication with a greater number of participants, preferably, or alternatively greater analytical detail will be included in this study and related meta-analysis.

### Data extraction

Data collection from relevant studies will be standardized using a data extraction template. The template has been informed by the recommendations made in the Cochrane Handbook of Systematic Reviews of Interventions [20], which was adapted to reflect this review’s focus on non-intervention studies. Data collected will include the lead author, study country, year of publication, study design, population studied, exposure (including the definition of recurrence), and outcome evaluated, number of cases, number of non-cases, the association measure, point estimate and 95% confidence intervals (CI), and any adjustment/stratification/matching variables [17]. Furthermore, there will be an open-text field to include any additional comments. In cases of missing or incomplete information in the included studies, we will contact study authors for further information, re-contacting after 2 weeks if no response is received. Where English language versions of publications are required, members of the review team will have the article translated.

### Data synthesis and statistical analysis

#### Exposure analysis subgroups

In order to ensure comparability and minimize heterogeneity in exposure measures, the different exposure subgroups will be considered in several ways in the meta-analyses of results. They are summarized in Table 3.

**Table 3:** The proposed different categorizations of the three forms of pregnancy loss

**Miscarriage**	A history of miscarriage: one or more miscarriages	Recurrent miscarriage: multiple miscarriages, defined in publications as a history of 2+ or 3+ miscarriages	Dose-response relationship: the number of miscarriages categorized as 0, 1, 2, and 3 or more
**Stillbirth**	A history of stillbirth: one or more stillbirths	Recurrent stillbirth: multiple stillbirths, defined in publications as a history of 2+ or 3+ stillbirths	Dose-response relationship: the number of stillbirths categorized as 0, 1, 2, and 3 or more
**Abortion**	A history of abortion: one or more abortions	Recurrent abortion: multiple abortions, defined in publications as a history of 2+ or 3+ abortions	Dose-response relationship: the number of abortions categorized as 0, 1, 2, and 3 or more

In all cases, the analyses will be conducted if sufficient studies are identified

All exposures will be compared with control groups without a history of the particular exposure in question. Where studies are deemed eligible for inclusion, but do not provide the required association measure, the raw data available in the study will be used to calculate an unadjusted estimate.

#### Outcome analysis subgroups

Both fatal and non-fatal occurrences of the following outcomes are eligible: CVD, CHD, MI, ischemic stroke, hemorrhagic stroke, and transient ischemic attacks. Given the hierarchical nature of some of these outcomes, meta-analyses will evaluate grouped outcomes:

1. CVD: all cardiovascular disease unless reported as a subtype only.
2. CHD: incorporating any individuals with diagnoses of CHD, MI, and individuals with a history of Coronary Artery Bypass Graft.
3. Cerebrovascular disease: incorporating ischemic stroke and hemorrhagic stroke.

A narrative synthesis of results will be presented, with the findings of individual studies reported both in tables and text. Meta-analyses will be conducted if three or more studies are found assessing a particular exposure-outcome combination. Given previous meta-analyses, we anticipate variability between included studies; therefore, a random-effects inverse variance-weighted model will be used to combine reported measures of association to produce a pooled odds ratio with 95% CI. The results will be displayed in forest plots separately for adjusted and unadjusted meta-analyses.

Between-study heterogeneity will be assessed using the *I*^2^ statistic and the Cochrane *χ*^2^ statistic [20]. *I*^2^ values of ≤ 50%, 50–75%, and 75–100% will be considered low, moderate, and high, respectively. If moderate or high levels of between-study heterogeneity are identified in meta-analyses of more than six studies, then meta-regression will be conducted to assess the relationship between the magnitude of risk and study characteristics of interest, including the age of participants, duration of follow-up, and year of study [27].

Small study effects will be evaluated by funnel plots and Egger’s test for meta-analyses that include six or more studies [20]. The Cochrane Handbook recommends at least ten studies to be included in a funnel plot; therefore, we acknowledge that there may be insufficient power to detect real asymmetry from chance [20]. Based on previous reviews, it is likely that all meta-analyses will include less than ten studies; therefore, we have chosen a more lenient cut-off, whilst being mindful not to over-interpret the results.

If there is evidence of funnel plot asymmetry and indication of significant bias from the Egger’s test, the trim-and-fill method will be used to correct for the asymmetry [28].

A number of sensitivity analyses will be considered. The first analysis will exclude studies with the largest effect estimates to assess the impact of these studies on the magnitude of the pooled result and the observed heterogeneity. The second analysis will include all studies and will re-run each meta-analysis with fixed-effects models to assess the consistency of the results.

Stratified analyses will also be considered where there are at least two studies in each strata. These analyses will stratify by (1) different levels of adjustment, (2) the population studied (e.g., N. American vs European), and (3) the level of bias in individual studies (as assessed by Newcastle-Ottawa Scale [NOS]).

*P* values of less than 0.05 will be considered to be significant. Analyses will be completed in Stata version 15 (StataCorp, College Station, TX, USA).

### Data quality and strength of evidence

Methodological quality and risk of bias within each of the included studies will be evaluated using the validated NOS for assessing the internal validity of case-control or cohort studies [29]. The NOS was chosen as it can assess the quality of both case-control and cohort studies, providing a comparable measure of quality for both study designs. The NOS uses a semi-quantitative scale for assessing the quality of non-randomized studies and allocates a maximum of nine stars to a study, across three categories. The categories are the quality of studies on the participant selection criteria, comparability of cases and controls, and exposure assessment (for case-control studies) or outcome assessment (for cohort studies).

The strength of the evidence for each exposure-outcome association will be assessed using the GRADE approach. This approach will assess the study limitations, providing an overall rating of the confidence in the results of each exposure-outcome association [30].

## Discussion

To our knowledge, this systematic review will be the first in the field to consider the full breadth of literature regarding the association between pregnancy loss and future maternal CVD. This protocol will add to the collective literature on systematic review protocols, of which there are few specifically addressing women’s health and CVD. This topic has its own unique challenges, including formulating precise inclusion and exclusion criteria that define pregnancy loss subtypes accurately. In particular, the gestational age boundaries for stillbirth, abortion, and miscarriage have changed over time [31]. Careful consideration is also required for the synthesis of data in which the exposure was reported in different categories (for example: 0 vs 1+; and 0, 1, 2, vs 3+). In order to accommodate potential differences, a narrative as well as a quantitative approach has been proposed to ensure all findings are reported.

Our proposed review has a number of strengths. It builds upon several sets of guidelines (MOOSE, PRISMA, Cochrane Handbook, and the Guidance on the Conduct of Narrative Synthesis in Systematic Reviews). Its novel scope has importance for academics, clinicians, and individual patients. The results of this review have the potential to inform the development of future risk assessment tools, such as the QRISK3 scoring tool [32], and aid doctors and patients in making informed clinical choices.

However, limitations are also anticipated. Although, stringent inclusion and exclusion will be applied, with a view to reducing between-study heterogeneity, heterogeneity is expected and will be assessed. Potential sources of heterogeneity include (1) differences in obtaining exposure information, such as self-reported vs medically verified pregnancy loss; (2) inconsistent or insufficient adjustment for confounding factors, including medical, psychosocial, and lifestyle factors such as depression, smoking, obesity, and hypertension; (3) population differences in the availability of abortions and the social acceptability of reporting them; and (4) differences in follow-up duration, if the magnitude of risk varies by time since the pregnancy loss.

Any protocol changes to address unforeseen limitations will be made through group discussion, and the date and rationale for the changes will be recorded in PROSPERO. Results of this systematic review, and associated meta-analysis, will be disseminated through publication in a relevant, peer-reviewed journal, presented at conferences, and disseminated to the public through media outlets.

## Data Availability

Not applicable.

## Appendix

**Table 4:** PRISMA-P Checklist

Section and topic	Item No	Checklist item	Page number
**Administrative information**			
Title			
Identification	1a	Identify the report as a protocol of a systematic review	1
Update	1b	If the protocol is for an update of a previous systematic review, identify as such	2
Registration	2	If registered, provide the name of the registry (such as PROSPERO) and registration number	1
Authors			
Contact	3a	Provide name, institutional affiliation, e-mail address of all protocol authors; provide physical mailing address of corresponding author	1
Contributions	3b	Describe contributions of protocol authors and identify the guarantor of the review	7
Amendments	4	If the protocol represents an amendment of a previously completed or published protocol, identify as such and list changes; otherwise, state plan for documenting important protocol amendments	n/a
Support			
Sources	5a	Indicate sources of financial or other support for the review	7
Sponsor	5b	Provide name for the review funder and/or sponsor	n/a
Role of sponsor or funder	5c	Describe roles of funder(s), sponsor(s), and/or institution(s), if any, in developing the protocol	7
**Introduction**			
Rationale	6	Describe the rationale for the review in the context of what is already known	2
Objectives	7	Provide an explicit statement of the question(s) the review will address with reference to participants, interventions, comparators, and outcomes (PICO)	2-3
**Methods**			
Eligibility criteria	8	Specify the study characteristics (such as PICO, study design, setting, time frame) and report characteristics (such as years considered, language, publication status) to be used as criteria for eligibility for the review	3-4
Information sources	9	Describe all intended information sources (such as electronic databases, contact with study authors, trial registers or other grey literature sources) with planned dates of coverage	3-4, 5
Search strategy	10	Present draft of search strategy to be used for at least one electronic database, including planned limits, such that it could be repeated	Table 3
Study records			
Data management	11a	Describe the mechanism(s) that will be used to manage records and data throughout the review	3-4
Selection process	11b	State the process that will be used for selecting studies (such as two independent reviewers) through each phase of the review (that is, screening, eligibility and inclusion in meta-analysis)	3-4
Data collection process	11c	Describe planned method of extracting data from reports (such as piloting forms, done independently, in duplicate), any processes for obtaining and confirming data from investigators	3-4
Data items	12	List and define all variables for which data will be sought (such as PICO items, funding sources), any pre-planned data assumptions and simplifications	3-4
Outcomes and prioritization	13	List and define all outcomes for which data will be sought, including prioritization of main and additional outcomes, with rationale	3-4
Risk of bias in individual studies	14	Describe anticipated methods for assessing risk of bias of individual studies, including whether this will be done at the outcome or study level, or both; state how this information will be used in data synthesis	5
Data synthesis	15a	Describe criteria under which study data will be quantitatively synthesised	4
	15b	If data are appropriate for quantitative synthesis, describe planned summary measures, methods of handling data and methods of combining data from studies, including any planned exploration of consistency (such as I^2^, Kendall’s τ)	4-5
	15c	Describe any proposed additional analyses (such as sensitivity or subgroup analyses, meta-regression)	4-5
	15d	If quantitative synthesis is not appropriate, describe the type of summary planned	4
Meta-bias(es)	16	Specify any planned assessment of meta-bias(es) (such as publication bias across studies, selective reporting within studies)	4-5
Confidence in cumulative evidence	17	Describe how the strength of the body of evidence will be assessed (such as GRADE)	GRADE

## Abbreviations

CHD: Coronary heart disease
CI: Confidence interval
CVD: Cardiovascular disease
GRADE: Grades of Recommendation Assessment, Development and Evaluation
MI: Myocardial infarction
MOOSE: Meta-Analyses Of Observational Studies in Epidemiology
NOS: Newcastle-Ottawa Scale
PRISMA: Preferred Reporting Items of Systematic Reviews and Meta-Analysis
PRISMA-P: Preferred Reporting Items of Systematic Reviews and Meta-Analysis for Protocols
